# Targeted genome editing in polyploids: lessons from *Brassica*

**DOI:** 10.3389/fpls.2023.1152468

**Published:** 2023-06-20

**Authors:** Niaz Ahmad, Samia Fatima, Muhammad Aamer Mehmood, Qamar U. Zaman, Rana Muhammad Atif, Weijun Zhou, Mehboob-ur Rahman, Rafaqat Ali Gill

**Affiliations:** ^1^ National Institute for Biotechnology and Genetic Engineering College, Pakistan Institute of Engineering and Applied Sciences (PIEAS), Faisalabad, Pakistan; ^2^ Department of Bioinformatics & Biotechnology, Government College University Faisalabad, Faisalabad, Pakistan; ^3^ Hainan Yazhou Bay Seed Laboratory, Sanya Nanfan Research Institute of Hainan University, Sanya, China; ^4^ College of Tropical Crops, Hainan University, Haikou, China; ^5^ National Center of Genome Editing, Center of Advanced Studies, Agriculture and Food Security, University of Agriculture, Faisalabad, Pakistan; ^6^ Department of Plant Breeding and Genetics, University of Agriculture Faisalabad, Faisalabad, Pakistan; ^7^ Ministry of Agriculture and Rural Affairs Key Lab of Spectroscopy Sensing, Institute of Crop Science, Zhejiang University, Hangzhou, China; ^8^ Key Laboratory for Biology and Genetic Improvement of Oil Crops, Ministry of Agriculture and Rural Affairs, Oil Crops Research Institute, Chinese Academy of Agricultural Sciences, Wuhan, China

**Keywords:** genome editing, CRISPR, *Brassica*, polyploid crops, crop improvement

## Abstract

CRISPR-mediated genome editing has emerged as a powerful tool for creating targeted mutations in the genome for various applications, including studying gene functions, engineering resilience against biotic and abiotic stresses, and increasing yield and quality. However, its utilization is limited to model crops for which well-annotated genome sequences are available. Many crops of dietary and economic importance, such as wheat, cotton, rapeseed-mustard, and potato, are polyploids with complex genomes. Therefore, progress in these crops has been hampered due to genome complexity. Excellent work has been conducted on some species of *Brassica* for its improvement through genome editing. Although excellent work has been conducted on some species of *Brassica* for genome improvement through editing, work on polyploid crops, including U’s triangle species, holds numerous implications for improving other polyploid crops. In this review, we summarize key examples from genome editing work done on *Brassica* and discuss important considerations for deploying CRISPR-mediated genome editing more efficiently in other polyploid crops for improvement.

## 1 Introduction

*Brassica* is an important genus of the *Brassicaceae* family, also known as Cruciferae or the mustard family. It is the 5^th^ largest monophyletic family among the angiosperms (Mun et al., 2009). The renowned *Brassica* “U’s triangle” (Nagaharu and Nagaharu, 1935) comprises three diploid species, including *Brassica rapa* (AA), *B. nigra* (BB), *B. oleracea* (CC), and their derivatives, three allotetraploid species, namely *B. napus* (AACC), *B. juncea* (AABB), and *B. carinata* (BBCC) (Cheng et al., 2017). *Brassica* crops are an important source of vegetable oils (Lu et al., 2018) and contribute significantly to global vegetable crop production, providing protein-rich feed for animals, as well as raw materials for biofuel production (Yang et al., 2018). Typically, *Brassica* seed contains 40–48% oil and 38–40% protein in the oil-free seed meal (Rahman et al., 2013). Oil extracted from *Brassica* species is considered one of the healthiest edible oils due to its low saturated fat contents (~7%), with the remaining 93% comprised of mono and poly-unsaturated fatty acids (Orsavova et al., 2015; Sharafi et al., 2015).

Polyploidy is a heritable state where a crop acquires one or more additional sets of genomes, either similar to the earlier one (autopolyploidy) or different ones (allopolyploidy), during the course of its evolution (Mason and Wendel, 2020). Positive consequences of polyploidy include gene redundancy for masking the effect of deleterious alleles, neo-functionalization of duplicated genes, emergent self-fertilization, fixed heterosis, and the capability of asexual reproduction, all of which have contributed significantly to the success of polyploid crops (Comai, 2005). Polyploidization has played a crucial role in the domestication of crop species through the creation of novel gene functions, combinations, interactions, and epigenetic changes, resulting in the enhanced adaptability of these crop species (Udall and Wendel, 2006). The beneficial effects of polyploidy are well elucidated, such as higher photosynthetic rates in polyploid species compared to diploids (Coate et al., 2012), increased fibre quality of allotetraploid cotton (Jiang et al., 1998), and grain hardness in tetra- and hexaploid wheat (Chantret et al., 2005).

Polyploidy is a significant contributor to speciation in plants, and almost every plant species displays signs of polyploidization. While polyploidy can create new variations that enhance a plant’s genetic potential, polyploid crops experience significant genome rearrangements, including multiple gene homologues, extensive patches of repetitive DNA, and high levels of heterozygosity – resulting in highly complex genomes. As a result, genetic improvement of field crops has become increasingly challenging (Xiong et al., 2011; Chen et al., 2019). However, the recent emergence of precise genome editing tools has revolutionized plant biotechnology. Genome editing entails creating site-specific mutations in the genome, such as insertions, deletions, or base substitutions (Manghwar et al., 2019). All genome editing tools, including Mega nucleases (MNs), Zinc finger nucleases (ZFNs), Transcription activator-like effector nucleases (TALENs), and Clustered Regularly Interspaced Short Palindromic Repeats (CRISPR), operate by creating double-stranded breaks (DSBs) followed by DNA repair via either through intrinsic NHEJ (non-homologous end joining) or homologous recombination (HR) with the provision of an extrinsic DNA fragment (Symington and Gautier, 2011; Ahmad et al., 2020).

The CRISPR system has completely transformed the genome editing landscape and has been extensively used in humans, animals, and plants for various applications, such as base editing, epigenetic modifications, prime editing, multiplexed genome editing, gene regulation, gene replacement, and labelling of specific chromosomal segments (Larson et al., 2013; Li et al., 2013; Nekrasov et al., 2013; Sander and Joung, 2014; Schaeffer and Nakata, 2015; Gallego-Bartolomé et al., 2018; Lowder et al., 2018; Anzalone et al., 2019; Wang et al., 2019; Zhang et al., 2019b). Compared to previous protein-DNA-based genome editing systems, CRISPR technology is cost-effective, robust, simple, and able to target multiple genes simultaneously (Cong et al., 2013; Shan et al., 2013). In plants, CRISPR technology has primarily been used to eliminate gene products that negatively affect plant traits through small indels, resulting in frame-shift mutations or premature stop codons (Ahmad and Mukhtar, 2017; Zhang et al., 2018b). CRISPR/Cas type II is the most widely used genome editing system (Hsu et al., 2014), consisting of two main components: Cas9 and guide RNA (gRNA). Cas9 is the nuclease responsible for creating DSBs at the target site, while gRNA is a chimeric RNA molecule formed by the homologous pairing of Precursor-CRISPR RNA (pre-crRNA) and Trans-activating RNA (tracrRNA) after transcription (Kumar et al., 2019; El-Mounadi et al., 2020). The gRNA contains 20 nucleotides that are complementary to a specific region in the target DNA, followed by a 3-bp consensus PAM (protospacer adjacent motif) sequence. The 3’ end of gRNA forms a loop structure, which helps fix the guide sequence to the target DNA, and interacts with Cas9 protein to form a ribonucleoprotein (RNP) complex responsible for creating DSBs at the target DNA location (Deltcheva et al., 2011).

The world’s population is expected to reach 10 billion by 2050, necessitating a significant increase in global food production (Hickey et al., 2019). To meet this challenge, various international organizations, including the UN and WHO, have called for a 60–70% increase in food production by 2050 (van Dijk et al., 2021). The improvement of *Brassica* crops could help meet these demands, as they are consumed in a variety of ways by both humans and animals and have many applications in industry. However, these crops face many challenges, including susceptibility to heat, cold, drought, shattering, insect/pests, deadly blackleg disease, and late maturation in the case of the widely cultivated *B. napus* (Augustine et al., 2014; Raman et al., 2014; Zhu et al., 2016; Wrucke et al., 2019; Kourani et al., 2022). *B. juncea*, another *Brassica* crop, is early maturing and tolerant to harsh climatic conditions but it contains high levels of anti-nutrients such as erucic acid and glucosinolates (Nour-Eldin et al., 2017). Therefore, the development of need-based superior *Brassica* crops that offer better yield potential, resilience to harsh climatic conditions, disease and insect pest resistance, and meet either edible or non-edible requirements has been a long-standing objective in *Brassica* breeding, which can be achieved more effectively with advanced biotech tools like CRISPR technology. The focus of this article is to review the latest research on the use of CRISPR technology for improving *Brassica* crops. This article also highlights the lessons learned from *Brassica* genome editing that can be applied to improve polyploid crops through CRISPR.

## 2 Strategies for crop improvement

Earlier efforts to improve *Brassica* involved traditional breeding approaches. However, due to its polyploidy, achieving breeding objectives through traditional methods has been quite challenging (Mason and Snowdon, 2016). Modern genetic tools have become necessary to develop high-yielding crop varieties on a sustainable basis against changing climates, given the complex genome of polyploids like *Brassica*. In cases where genetic variability is not present in the crossable germplasm for a specific trait, spontaneous natural mutations or induced mutagenesis can also be an option. However, finding the desired edits can be complicated and time-consuming, and sometimes the desired mutations may negatively impact the desired characteristics such as yield. As a result, breeders have to undergo multiple laborious and time-taking crossing and selfing procedures to combine mutant copies in a single line (Schouten and Jacobsen, 2007).

**Figure 1:**
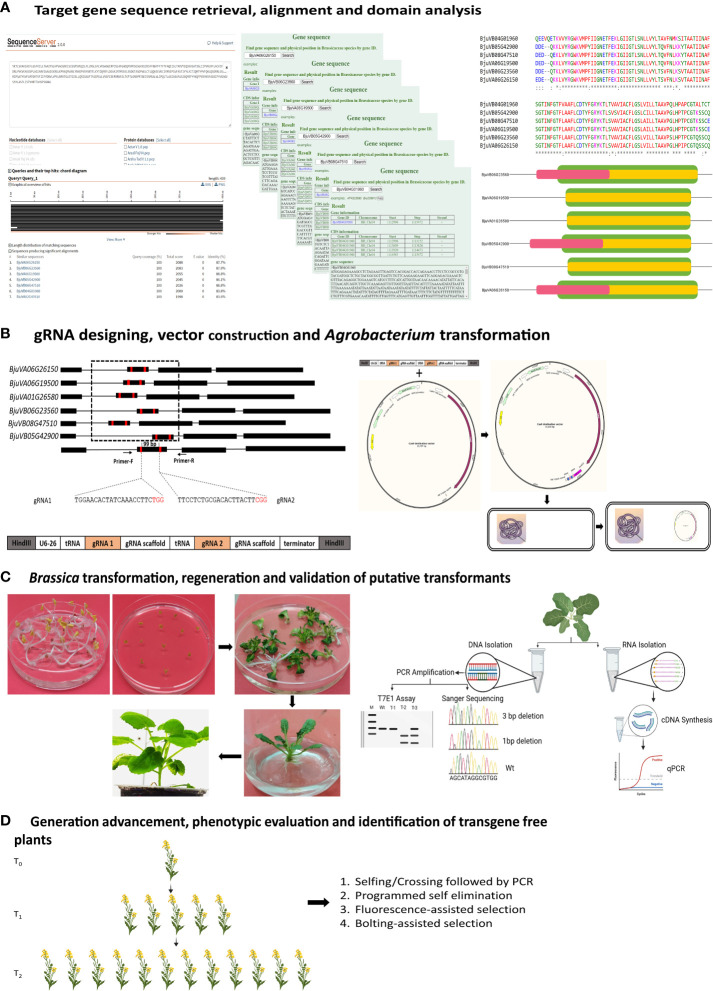
Schematic illustration of the steps involved in the development of transgene-free genome-edited *Brassica* plants. **(A)** shows the identification and retrieval of target gene sequences from public databases, such as GenBank or The Brassicaceae Database (BRAD V3.0; http://brassicadb.cn). The sequences are aligned to identify potential domains for designing gRNA. **(B)** shows the gRNA designing, construction of plant expression vectors followed by transformation of that construct into *Agrobacterium* strain for plant transformation. **(C)** represents Agrobacterium¬- mediated *Brassica* transformation using cotyledonary explants, shoot regeneration on the selection medium, root induction, establishment of the plants in compost, and screening of the putative transformants using techniques such as T7 endonuclease I assay, Sanger sequencing, or qPCR. **(D)** illustrates the process of eliminating marker genes to obtain transgene-free edited plants through Mendelian segregation. Transgene-free edited plants can also be obtained by programmed self-elimination, using suicidal genes, and can be immediately identified using fluorescent genes. Another way of identification of transgene-free edited plants is by incorporating genes that confer distinct phenotypes, such as early flowering (Bolting assisted selection) or fluorescence. Some parts of the picture were created using BioRender.com.

An alternative approach to creating desired traits in field crops is genetic engineering. This approach has been used to engineer several desirable traits in plants, including herbicide tolerance (Blackshaw et al., 1994; Rong et al., 1997; Qing et al., 2000), oil quality improvement (Stoutjesdijk et al., 2000; Liu et al., 2001; Naeem et al., 2020), insect/pest resistance (Kanrar et al., 2002; Wang et al., 2005; Liu et al., 2011; Rani et al., 2017), salt tolerance (Prasad et al., 2000; Zhang et al., 2001), cold tolerance (Jaglo et al., 2001), shattering resistance (Østergaard et al., 2006), phytase enzyme production (Ponstein et al., 2002; Wang et al., 2013), and development of male sterile and fertility restorer transgenic lines (Jagannath et al., 2001; Jagannath et al., 2002). However, genetic transformation approaches are associated with health and environmental concerns, and genetically modified crops have low acceptance by the public (See Ahmad and Mukhtar, 2017 for review). The controversy surrounding the development of transgenic plants through conventional genetic engineering approaches has led to the ban of open-field cultivation in many countries, such as France, Germany, Greece, Italy, Turkey, Ecuador, Russia, Madagascar, Netherlands, Algeria, Saudi Arabia, Venezuela, Ukraine, Austria, Hungary and Poland (Jaganathan et al., 2018). Genome editing tools, particularly CRISPR, allow the development of transgene-free edited plants that can pass the regulatory framework easily and increase public acceptance of GM crops (**Figure 1**). Consequently, CRISPR has become a tool of choice for developing edited plants with desired traits (Chen and Gao, 2014). In the following sections, we will discuss different studies reporting the successful deployment of the CRISPR/Cas platform for the improvement of *Brassica.*

### 2.1 Yield improvement

Yield is a complex trait that depends on several parameters including plant height, number of siliques, number of branches, multilocular siliques, and seed shattering. Researchers have targeted these primary traits to increase yield. For instance, when a gene *ALCATRAZ* (*ALC*) involved in valve margin development, was edited, not only was pod-shattering reduced, but the silique length was also significantly improved (Braatz et al., 2017). Likewise, editing the *CLAVATA* (*CLV1*, *CLV2* and *CLV3*) genes involved in multilocular silique phenotype by regulating stem cell function, cell fate, and proliferation in *Brassica* increased the number of seeds as well as the seed weight (Yang et al., 2018). Repressing all five copies of *JAGGED* (*JAG*) genes in *B. napus* resulted in disturbed plant phenotypes, such as unorganized cell identity in floral primordia leading to pod deformation and serrated leaves. Transgenic plants with edits in *BnJAG.A08* showed alterations in the dehiscence zone development only, with increased replum area, decreased pod length and improved pod shattering resistance (Zaman et al., 2019a). Sriboon et al. (2020) edited all five copies of *BnaTFL1*, a flowering inhibitor gene also implicated in the regulation of plant architectural traits. The authors showed that plants with edits in all gene copies displayed significant changes in the plant architecture, such as reduced plant height, branch initiation height, branch number, silique number, and the number of seeds/siliques on the main inflorescence. The most severe alterations in the plant architecture were observed in the *BnaC03.tfl1* edited plant. This suggests that *BnaC03.TFL1* not only plays a significant role in flowering mechanism but also plant architecture traits along with other *BnaTFL1* gene homologues. In a recent study, Khan et al. (2021) edited four homologues of *Brassica napus* gene *BnaEOD3*, resulting in plants with shorter siliques, with a smaller yet increased number of seeds/siliques. The study further illustrated the differential and quantitative involvement of *BnaEOD3* gene homologues in seed development. For instance, *BnaC04.EOD3b* and *BnaA04.EOD3* were found to play the most significant and minor role in seed development, respectively.

### 2.2 Plant architecture traits

Plant architecture traits such as plant height, branch number, and root architecture are associated with yield. Therefore, understanding the function of genes governing these traits can help improve them and ultimately increase yield. Lawrenson et al. (2015) edited the *GIBBERELLIN 3-OXIDASE1* (*BolC.GA4.a*) gene in *B. oleracea* and found it to be involved in regulating the gibberellic acid pathway. Kirchner et al. (2017) investigated the role of the *BcFLA* gene in *B. carinata* and found that edited plants developed shorter root hairs. Leaf morphology is an important trait that indirectly contributes to yield increase by affecting evapotranspiration rates, sunlight penetration, and insect/pest preference. In *Brassica* species, diverse leaf morphology is observed, including entire, serrated and lobed leaves. The lobed leaf phenotype is preferred for high-density crop cultivation, mechanized harvesting, and reduced insect and disease incidence. Hu et al. (2018) studied the role of the *BnA10.LMI1* gene in the formation of lobed leaves in *B. napus* and reported that the *LMI1* gene encodes for an HD-Zip I transcription factor that regulates the expression of the *LLA10* gene, which is responsible for the lobed leaf shape. Zheng et al. (2020) developed edited plants with mutations in *BnaMAX1*. The resulting plants displayed a semi-dwarf phenotype with enhanced branching and siliques, resulting in a significant yield increase. Cheng et al. (2021) used an A3A-PB3 base editing system to replace C with T in *BnRGA* and *BnIAA7* genes separately and obtained dwarf plant types in both cases, suggesting that both of these genes are involved in regulating plant height. Stanic et al. (2021) edited the *BnD14* gene and observed a significant increase in the number of branches (200%) and the number of flowers per plant (37%), suggesting it is a single major potential candidate gene for improving yield-related traits. Fan et al. (2021) developed semi-dwarf *B. napus* plants with compact architecture and without any undesirable traits by targeting *BnaA03.BP* gene, concluding that *BnaA03.BP* alone could be a potential candidate for a moderate phenotype.

### 2.3 Quality improvement

Improvement in total oil content, along with a better fatty acid profile, is a major goal in improving oilseed *Brassica* crops. The manipulation of these traits has been achieved through the successful application of CRISPR/Cas9 technology. Studies have shown that yellow seed coat colour is associated with enhanced oil content and better fatty acid composition in *Brassica* species. Double knockout yellow seed phenotypes were produced with enhanced seed oil (51.8%) and protein content (16.97%) with a modified fatty acid profile without causing any severe defects in yield-related traits by targeting *TRANSPARENT TESTA8* (*BnTT8*) (Zhai et al., 2020). Similarly, Xie et al. (2020) edited the *TRANSPARENT TESTA2* (*BnTT2*) gene in *B. napus*, resulting in a yellow-seeded phenotype with improved oil content up to 45–47% and better fatty acid composition with enhanced linoleic and linolenic acid.

The nutritional quality of the oil is primarily determined by the proportion of different fatty acids present. CRISPR technology has also been used to develop *Brassica* plants with a better fatty acid profile. Oleic acid is a monounsaturated acid that not only increases the shelf life of vegetable oil but also protects it from damage due to oxidation. High oleic acid *B. napus* lines were produced by introducing a novel edited allele of the *FATTY ACID DESATURASE 2* (*FAD2*) gene involved in the conversion of oleic acid into linoleic acid (Okuzaki et al., 2018; Huang et al., 2020).

In addition to being rich in oil contents, *Brassica* seeds are also a good source of proteins with a good amino acid profile. However, the presence of certain anti-nutrients called glucosinolates reduces the utilization of seed meal as animal feed. Neequaye et al. (2021) targeted three gene copies of the *MYB28* gene involved in the regulation and biosynthesis of aliphatic glucosinolates in *B. oleracea*. Editing was observed in only C2 and C9 copies of the gene but not in the C7 gene copy. The knockout plants showed notable downregulation of aliphatic glucosinolates biosynthesis and accumulation in the leaves and florets of the edited plants. This implies that a significant reduction in the aliphatic glucosinolates accumulation can be achieved by editing two of the three gene copies and the C7 copy may have a subfunctional role in glucosinolates accumulation, or it may evolve into a pseudogene in the course of evolution.

Phytic acid is another important anti-nutrient present in *Brassica* seed that blocks the absorption of minerals and proteins in monogastric animals. Sashidhar et al. (2020) edited the members of the BnITPK gene family (four from BnITPK1 and two from the BnITPK4 sub-family) involved in the biosynthesis of phytic acid. They observed a significant reduction (~35%) in the phytic acid content in edited lines.

*Brassica* seed, particularly those of yellow mustard (Sinapis alba) and brown mustard (B. juncea), contains proteins that can trigger allergic reactions, such as urticaria, wheezing, abdominal pain, dyspnea, and life-threatening anaphylactic shocks (Matsuo et al., 2015). One of the proteins, BrajI, found in *B. juncea* seeds, cause allergic reactions. The development of allergen-free food could offer a reliable solution to this issue. Assou et al. (2022) employed CRISPR/Cas9 genome editing in *B. juncea* and successfully eliminated BrajI from the seeds.

### 2.4 Biotic and abiotic stress tolerance

Biotic and abiotic stresses are major factors that depress yield in field crops, including *Brassica*. The CRISPR platform is increasingly being used to develop crops that are resilient to these stresses. *Sclerotinia sclerotiorum* is a fungal disease that causes significant yield losses in *Brassica*. Sun et al. (2018) edited *BnWRKY11* and *BnWRKY70* genes in *B. napus*. The lines with edited *BnWRKY70* showed enhanced resistance to *S. sclerotiorum* while those with *BnWRKY11* showed no significant difference. Verticillium longisporum (Vl43) is another serious fungal disease of *Brassica* responsible for significant yield losses due to the absence of effective genetic resistance in the crop. Pröbsting et al. (2020) developed knock-out plants of *B. napus* for the BnCRT1 gene using CRISPR and observed a significant drop in the fungal susceptibility of the crop against Vl43. Zhang et al. (2022a) elucidated the mechanism of rapeseed resistance against S. sclerotiorum using CRISPR/Cas9 technology. They identified a positive role of MITOGEN-ACTIVATED PROTEIN KINASES, *BnaA03.MKK5-BnaA06.MPK3/BnaC03.MPK3* module in resistance against S. sclerotiorum. The double knock out plants showed an enhanced susceptibility to the disease.

Wu et al. (2020) mutated BnaA6.RGA and the resulting plants showed increased drought tolerance. However, the quadruple bnarga edited plant line exhibited reduced drought tolerance.

Mineral deficiencies, such as boron, are significant factors involved in stunted growth and yield reduction in Brassica. However, the mechanism involved in the adaptation of plants in low boron environments is not clear. Feng et al. (2020) elucidated the function of the BnWRKY47 transcription factor under boron stress using CRISPR technology. They found that BnWRKY47 was responsible for the adaptation of *B. napus* to low boron environments by up-regulating the boric acid channel gene BnaA3.NIP5;1.

As biotic and abiotic stresses cause major yield losses in almost all major field crops including polploid crops, therefore, utilizing CRISPR could be an effective strategy for developing stress-tolerant crops.

### 2.5 Insights into functional genomics

In polyploid crop species, the most important traits are controlled by multiple genes, making it necessary to identify and select genes that significantly contribute to the trait of interest for manipulation. In polyploid crops like *Brassica*, the situation becomes more complicated due to the presence of many copies of a single gene in the genome and their functional redundancy. Thus, functional genomics and proper validation of target genes is crucial for improving particular traits. Genes that control a trait can be divided into two classes: structural (protein coding) and regulatory (transcription factors). Structural genes, with a direct role in crop traits, provide an attractive target for precise genome editing. Regulatory genes, mostly consisting of transcription factors and other regulatory elements, are involved in regulating structural genes. They are induced by certain biological processes such as biotic and abiotic stress signals, and hence, control the expression of many downstream structural genes. Therefore, targeting regulatory genes may help improve crops against complex stress phenomena.

CRISPR/Cas9 technology can be effectively employed to gain insights into the functional roles of specific genes by producing knockout or loss-of-function alleles. Pod shattering is a widely investigated subject in the case of the *Brassica* crop because of its significant role in yield reduction. Previously, *ALCATRAZ* (*ALC*) and *INDEHISCENT* (*IND*) gene homologues were found to have a significant role in pod dehiscence. Zhai et al. (2019) assessed the function of these genes in pod shattering in *B. napus* using CRISPR/Cas9 technology. The results from the phenotypic evaluation of homozygous knockout plants showed the conserved and essential role of the *BnIND* gene, whereas the *BnALC* gene showed limited potential for *B. napus* pod-shattering resistance. Additionally, *BnIND* gene homologues exhibited functional redundancy, with a major contribution from *BnA03.IND*.

The CRISPR platform has also been used in *Brassica* to carry out functional genomic studies on genes with multiple copies. For example, Zhang et al. (2019a) used CRISPR/Cas9 to understand the functions of *LYSOPHOSPHATIDIC ACID ACYLTRANSFERASE* (*LPAT2*) and (*LPAT5*) family genes, both of which have seven and five homologues, respectively. These genes encode several key enzymes in the Kennedy pathway, which catalyze the fatty acid chains into 3-phosphoglycerate and promote oil production in the form of triacylglycerols. The edited lines showed enlarged oil bodies but lesser oil content than the wild type, suggesting that the *LPAT2/5* gene families play an important role in fatty acid biosynthesis.

Heat stress severely impacts seed production in *Brassica* by altering the normal anatomy of floral organs. Unfortunately, the molecular mechanism underlying this phenomenon is poorly understood. One mutation produces novel pistil-like flowers in *B. rapa*, where four of the five sepals merge to form a ring structure that encapsulates abnormal stamens and a pistil, leading to poor seed production. The mutation is called sepal carpal modification (*scm*) and is found in the *BrAP2* gene homologues, which are orthologues of the *Arabidopsis APETALA (AP2)* gene. To investigate the function of four *BnAP2* gene homologues in sepal and petal development, rapeseed knockout plants were generated using the CRISPR/Cas9 tool (Zhang et al., 2018a). The quadruple knockout plants showed an *scm*-like appearance, confirming the functional conservation of the *AP2* gene in *Brassica*. This study also provides information on the modification of floral organs by genome manipulation for yield improvement (Zhang et al., 2018a). Jedličková et al. (2022) studied the function of the TRYPTOPHAN AMINOTRANSFERASE (BnaTAA1) gene in the auxin biosynthesis pathway.

Yang et al. (2017) simultaneously targeted 12 genes from different families, including the RGA, FUL, DA1, and DA2 gene families. Most of the plant lines exhibited a mutation frequency of 5.3% to 100%, with the development of homozygous and bi-allelic mutations stably inherited to successive generations, highlighting the fact that single gene editing in polyploid species did not yield any significant phenotypic differences. Xiong et al. (2019) functionally characterized the gene involved in pollen growth and development in *B. compestris*. They targeted three homologous *PECTIN METHYLESTERASE* (*PME*) genes using CRISPR/Cas9 technology and explored that *Bra003491* had a significant role in pollen growth and development, while the other two homologues, *BRA007665* and *Bra014410*, may function redundantly. Wang et al. (2022b) employed the CRISPR/Cas9 system to explore the function of STARCH BRANCHING ENZYME (BnaSBE) genes in starch structure and overall throughput of *B. napus*. The analysis revealed the least activity of the starch-binding enzyme, binding frequency, higher starch-bound phosphate content, and altered pattern of amylopectin chain length distribution in sextuple edited plants compared to the wild-type.

### 2.6 Flowering time and the flower colour

The development of early maturing *B. napus* has long been a breeding objective due to the crop’s ability to escape disease and aphid attack, both of which can significantly reduce crop yields. Jiang et al. (2018) investigated the role of methyl transferase in two *SET DOMAIN GROUP 8* (*BnSDG8.A* and *BnSDG8.C*) genes in rapeseed using CRISPR/Cas9 technology. The *SDG8* gene is pleiotropic in nature and regulates multiple biological processes, such as flowering time and plant height. The mutations significantly reduced flowering time compared to the wild type. Jeong et al. (2019) developed early-flowering *B. rapa* (Chinese cabbage) through targeted editing of the four homologous *FLOWERING LOCUS C* (*BrFLC*) genes, eliminating the need for vernalization for flower induction. In a similar effort to reduce flowering time, Sriboon et al. (2020) targeted five gene copies of *TERMINAL FLOWER 1* (*BnaTFL1*), a flowering inhibitor gene and also regulate plant architectural traits, using CRISPR/Cas9 technology. They obtained knockout plants for all gene copies and a knockout plant for *BnaC03.tfl1/BnaC09.tfl1*. However, only the single knockout *BnaC03.tfl1* and double knockout *BnaC03.tfl1/BnaC09.tfl1* displayed an early flowering phenotype, demonstrating the significant role of *BnaC03.TFL1* in the flowering mechanism. Vernalization treatment has been found to reduce the transcript levels of the *FLC* gene in *Arabidopsis*, thereby inducing flowering. Hong et al. (2021) targeted three gene homologues of *VERNALIZATION 1* (*VRN1*) using CRISPR/Cas9 technology to develop late flowering phenotypes in *B. rapa*. *VRN1* is known to be involved in the negative regulation of *FLC* gene expression. In an effort to attain early maturity, Ahmar et al. (2022) targeted *SHORT VEGETATIVE PHASE* (*BnaSVP*) genes using CRISPR. Homozygous transgene-free lines showed a significant decrease in flowering time, with the shortest flowering time in the quadruple edited plant as compared to that of the wild type, confirming the quantitative contribution of *BnaSVP* gene copies in the flowering time trait in a polyploid species.

*Brassica* crops are also consumed as vegetables, and cabbage (*B. oleracea* var. *capitata*) is well-known for its better nutritional profile, anti-cancer, and anti-inflammatory properties. However, early flowering affects the yield and quality of cabbage by reducing vegetative growth. To address this, Park et al. (2019) targeted two alleles of the flowering time regulator *BoGIGANTEA* (*GI*) gene using CRISPR, resulting in a significant increase in flowering time in the edited lines. Li et al. (2018) targeted five gene copies of *SQUAMOSA-PROMOTER BINDING PROTEIN-LIKE 3* (*BnSPL3*) gene, a key floral activator involved in the plant’s transition from vegetative to the reproductive stage, in oilseed rape. They obtained lines with all five edited gene copies that displayed significantly delayed phenotypes compared to wild-type control plants.

*Brassica* plants have also been cultivated as ornamental crops in some countries, such as China. Flower colour is an important aesthetic value of the crop, and Liu et al. (2020) developed phenotype with orange-coloured flowers by knocking out the *ZEAXANTHIN EPOXIDASE* (*BnaA09.ZEP* and *BnaC09.ZEP*) genes involved in the increased lutein content and decreased violaxanthin content in petals, specifically giving orange colour to petals.

### 2.7 Pod shattering

Pod shattering is a significant factor that reduces yield in *Brassica*. The *SHATTERPROOF (SHP1* and *SHP2*) genes regulate lignin content in the dehiscence zone, which contributes to seed shattering in *Brassica*. To address yield losses due to pod shattering, Zaman et al. (2021) targeted six *BnSHP1* and two *BnSHP2* homologues to develop edited lines with multiple edited gene copies. The phenotypic evaluation showed that *BnSHP1A09* may have a significant role in regulating lignin content in the dehiscence zone, with BnSHP1A09/C04-B/A04 and BnSHP2A05/C04-A showing the most reduction in lignin and separation layer adjacent to valves and replum. To confirm the functional redundancy of these genes, a single knockout line was crossed with a quadruple knockout line to develop a line with mutations at all five homologues, namely, BnSHP1A09, BnSHP1C04-B, BnSHP1A04, BnSHP2A05, and BnSHP2C04-A. The resulting five-homologues knockout plant showed significantly increased resistance to pod shattering.

### 2.8 Herbicide tolerance

*Brassica* yields are also affected by weed infestations, resulting in significant yield losses and quality deterioration. Effective weed management involves the use of herbicides, which is labour and cost-effective. However, *Brassica* crops tolerant to herbicides, developed through traditional genetic engineering approaches, fall under strict GMO regulations and hence cannot be cultivated in many countries. Wang et al. (2021) recently reported the development of glyphosate-tolerant *B. napus* using a CRISPR/Cas9-based geminiviral donor DNA replication system. They used a bacterial Cys4-based single RNA processing system to replace the endogenous *5-ENOLPYRUVYLSHIKIMATE-3-PHOSPHATE SYNTHASE* (*EPSPS*) gene, involved in the synthesis of branched amino acids, with its modified form. Additionally, Wang et al. (2022a) used the modified CRISPR/Cas9-based editing system to induce a point mutation (C to T) in the *ACETOLACTATE SYNTHASE* (*ALS*) gene, which encodes for a key enzyme involved in the biosynthesis of branched-chain amino acids, resulting in the creation of herbicide-tolerant cauliflower plants. The *ALS* gene presents a potential target site for several important herbicides. **Table 1** provides a summary of different traits edited in *Brassica* using CRISPR.

**Table 1:** CRISPR-mediated modification of traits in various *Brassica* crops.

Crop	Target trait	Target gene	No. of genes/homologues targeted	Number of gRNAs used	Type of mutation	Editing Efficiency in T_o_	Genome editing technology	Reference
1. Yield Related Traits								
*Brassica napus*	Valve margin development	*BnALC*	02	01	Knockout	N.A	CRISPR/Cas9	(Braatz et al., 2017)
	Multilocular siliques	*BnCLV1, BnCLV2, BnCLV3*	02 (for each single gene)	02 gRNAs for *BnCLV3* and 044 for each of *BnCLV1* and *BnCLV2*		0-48%		(Yang et al., 2018)
	Pod shattering	*BnJAGGED*	05	03		60%		(Zaman et al., 2019a)
	Seed production	*BnEOD3*	04	04		45%		(Khan et al., 2021)
2. Quality Related Traits								
*Brassica oleracea*	Glucosinolates biosynthesis	*BoMYB28*	03	04	Knockouts	N.A	CRISPR/Cas9	(Neequaye et al., 2021)
*Brassica napus*	Fatty acid desaturation	*BnFAD2*	01	02		5-50%		(Okuzaki et al., 2018)
			04	02		19%		(Huang et al., 2020)
	Seed coat colour	*BnTT8*	02	04		14%		(Zhai et al., 2020)
	Fatty acid modification	*BnLPAT2*	07	03		17-68		(Zhang et al., 2019a)
		*BnLPAT5*	04	01				
	Seed coat colour	*BnaTT2*	02	02		4%		(Xie et al., 2020)
	Phytic acid biosynthesis	*BnITPK*	03	02		N.A		(Sashidhar et al., 2020)
	Glucosinolates transport	*BnGTRs*	12	04		N.A		(Li et al., 2021)
*Brassica juncea*	Allergen protein Bra j i	*Bra j i*	02	08		47-50% 81-87%		(Assou et al., 2022)
3. Plant Architecture Traits								
*Brassica oleracea*	Plant height	*BolC.GA4.a*	01	02	Knockouts	10%	CRISPR/Cas9	(Lawrenson et al., 2015)
*Brassica carinata*	Root hair architecture	*BcFLA1*	01	02		N.A		(Kirchner et al., 2017)
*Brassica napus*	Leaf shape	*BnA10.LMI1*	01	04		N.A		(Hu et al., 2018)
	Plant height and branching	*BnMAX1*	02	02		56-67%		(Zheng et al., 2020)
		*BnD14*	02	04		N.A		(Stanic et al., 2021)
	plant height	*BnTFL1*	05	04		18%		(Sriboon et al., 2020)
	Plant height	*BnRGA*	*04*	01	Point mutation	25%	A3A-Cytidine deaminase base editor	(Cheng et al., 2021)
	Plant height	*BnIAA7*	*05*	01		31%		
4. Pod Shattering Resistance								
*Brassica napus*	Pod shattering	*BnSHP1*	06	04	Knockouts	22-35%	CRISPR/Cas9	(Zaman et al., 2021)
		*BnSHP2*	02	02				
		*BnIND*	02	04		N.A		(Zhai et al., 2019)
5. Flowering and Reproductive Traits								
*Brassica oleracea*	Flowering time	*BoGIGANTEA* (*GI*)	01	04	Knockouts	N.A	RNPs CRISPR/Cas9	(Park et al., 2019)
	Self-incompatibility	*BoSRK3*,	01	04		44%	CRISPR/Cas9	(Ma et al., 2019)
	Male sterility	*BoMS1*	01	04		33%		
*Brassica rapa*	Flowering time	*BraFLC*	04	07		97-100%		(Jeong et al., 2019)
	Pollen development	*BrPME*	03	03		20-56%		(Xiong et al., 2019)
	Flowering time	*BrVRN1a*	01	04		52%		(Hong et al., 2021)
*Brassica napus*	Vegetative to reproductive phase transition	*BnSPL3*	05	01		96-100%		(Li et al., 2018)
	Flowering time	*BnSVP*	04	04		45%		(Ahmar et al., 2022)
	Floral transition	*BnSDG8*	02	01		N.A		(Jiang et al., 2018)
	Flowering time	*BnTFL1*	05	04		18%		(Sriboon et al., 2020)
	Flower colour	*BnZEP*	02	04		N.A		(Liu et al., 2020)
	Flower shape	*BnAP2*	02	01		N.A		(Zhang et al., 2018a)
6. Biotic and Abiotic Stress Resistance								
*Brassica napus*	Sclerotinia sclerotiorum resistance	*BnWRKY11*	02	03	Knockouts	54%	CRISPR/Cas9	(Sun et al., 2018)
		*BnWRKY70*	04	03		50%		
	*Verticillium longisporum* resistance	*BnCRT1a*	04	01		20%		(Pröbsting et al., 2020)
	Boron stress	*BnWRKY47*	01	02		N.A		(Feng et al., 2020)
7. Herbicide Resistance								
*Brassica napus*	Herbicide resistance	*BnALS*	03	01	Point mutation	28%	A3A-Cytidine deaminase base editor	(Cheng et al., 2021)

NA, Not available.

## 3 Lessons from *Brassica*

CRISPR/Cas9 technology has immense potential and advantages, but it also poses significant challenges when it comes to genome editing of crops with complex polyploid genomes like *Brassica*. While discussing the successful deployment of CRISPR in various fields, it is important to consider the potential difficulties of editing the genomes of polyploid species with complex genomes.

### 3.1 Genome complexity

One of the primary challenges is genome complexity. Polyploid crops, including *Brassica* species, have undergone complex genomic rearrangements, including at least one whole genome duplication event in their evolutionary history. The triplication events undergone by *Brassica* species after separation from *Arabidopsis* resulted in highly complex genomes. For instance, Yang et al. (2018) found three gene copies of *BnCLV3* in the released *B. napus* genome while targeting *BnCLV* gene homologues, but they were able to identify only two gene copies in the pure line J9707. Similarly, in an attempt to edit *BnSBE2* gene homologues, Wang et al. (2022b) retrieved a total of six genes (four *BnSBE2.1* and two *BnSBE2.2)* from the released *B. napus* genome database, but they found only three gene copies of *BnSBE2.1* and one gene of *BnSBE2.2* in the cultivar DH12075. This high level of genome complexity and gene redundancy makes gene editing a challenging task in polyploid crops, particularly when dealing with recessive traits that require the elimination of all alleles (Khan et al., 2021; Assou et al., 2022). It is worth mentioning that for traits that require editing of all genes, as is the case with polyploid crops, the phenotype may quickly revert when grown in the field, particularly in open-pollinated species (Wang et al., 2014). The reversion frequency in the case of recessive alleles has been proposed to be 50% higher than dominant genes (Ahmad et al., 2020). Therefore, it is important to undertake preventive strategies such as introducing buffer zones and maintaining the original edited versions in isolated blocks.

### 3.2 Gene redundancy

The availability of complete, well-annotated, and elucidated genome sequence information, along with functional genomic studies, is a prerequisite for genome editing technology, as it facilitates the direct identification of candidate genes for editing. However, the highly repetitive and complex nature of polyploid genomes poses risks for computational biologists, such as phasing, full chromosome assembly, gene annotations, and differentiation between homo- and homeologues during the development of polyploid genome sequences (Kyriakidou et al., 2018). Additionally, chromosomal rearrangements and epigenetic modifications in polyploid plant species often result in transcriptional changes, including the activation of transposable elements, neo-functionalization of duplicated genes, and biased homeologues expression (Wendel et al., 2018), which can make it difficult to link genotype to phenotype and molecular characterization of edited genes in polyploid species. Due to a sequence resource deficit, and biased gene expression, genome sequencing is often limited to a model plant within a species. This can pose difficulties in selecting target genes, which can be addressed by using translational genomics approaches to develop genotype-phenotype connections (May et al., 2023).

The actual number of homologue and homeologue genes underlying a trait and their functionality status remains unclear in polyploid crops. However, gene dosage phenomena have been observed for several traits in polyploid crops, where each gene has a small contribution to the execution of a trait (Schaart et al., 2021).

### 3.3 gRNA designing

Designing gRNA is a critical step in CRISPR/Cas9 genome editing, as it needs to be highly specific to the target gene. In a polyploid crop, where multiple genes are targeted simultaneously using a single gRNA, specific gRNA design becomes a challenging task (Zaman et al., 2019b). Although various online tools have been developed for gRNA designing and predicting gRNA efficiency in polyploid crops, they may not guarantee the efficiency of a particular gRNA in living cells (Okada et al., 2019). Therefore, gRNA in polyploid crops like *Brassica* are manually designed after multiple sequence alignments on the conserved regions of all targeted gene copies. If a conserved region is not present in all the gene homologues, these homologues are grouped, and gRNAs are designed on the conserved region of each group (Zaman et al., 2019b). Apart from gRNA specificity, gRNAs with a GC content of 50–60% and a relatively shorter length of 18 bp, preferably starting from A or G, exhibit high on-target efficiencies (Feng et al., 2014; Tsai et al., 2015; Pan et al., 2016).

An important challenge for gRNA design is its strict dependence on PAM, which is NGG in the case of SpCas9 (Jinek et al., 2012). This PAM dependency limits the choice of a target site in the target gene. The problem is further exacerbated when designing gRNA in polyploid species. The development of altered or near PAM-less systems may present a much-needed solution to this challenge and can also enhance the utilization of CRISPR technology in functional genomics and precision breeding. Scientists have modified existing Cas enzymes and identified new Cas enzymes that have different sets of PAM sequences (Steinert et al., 2015). These newly discovered Cas enzymes include SpCas9 NG, VQR, and VRER versions, which recognize NG, NGA, and NGCG PAMs, respectively, while xCas9 3.7 can recognize GAT, GAA, and NG PAMs with increased specificity (Kleinstiver et al., 2015; Zhong et al., 2019). Walton et al. (2020) developed the Cas9 variant SpG with PAM NGN and further optimized it to create a near PAM-less Cas9 variant named SpRY (NRN and NYN), which can recognize almost all PAMs. Ren et al. (2021) utilized the SpRY nuclease and base editor in rice and developed herbicide-tolerant lines with high editing efficiency. The application of these near PAM-less Cas enzymes has been limited to the model species to date, such as rice and *Arabidopsis* (Endo et al., 2019; Yamamoto et al., 2019; Qin et al., 2020), and has not been reported in polyploid crops.

### 3.4 Off-target effects

Off-target effects pose a significant challenge to CRISPR-based genome editing as a whole. Although plants have comparatively fewer off-target effects compared to animals or humans, they can still lead to undesired changes in plant phenotype. Therefore, eliminating off-targets effects is crucial for precise and efficient genome editing. The gRNA should be highly specific to the target site, as non-specific gRNA can edit the non-target sites in the genome, resulting in unpredictable changes in the genome and phenotype. It has been reported that the number of off-targets increases with the number of gRNAs used, which is often the case in polyploid crops where editing of multiple copies is required. This may explain the high number of off-targets observed in *Brassica*. For example, in a study by Ahmar et al. (2022), using four gRNAs in a multiplexed editing approach for editing four gene homologues of the *BnaSVP* gene resulted in the gRNA targeting all the homologues having the most number of off-target sites. Zhai et al. (2019) found 26 off-target sites in total corresponding to three designed gRNAs targeting two genes. Assou et al. (2022) predicted 24 off-target sites in two edited lines in *B. juncea*, while Huang et al. (2020) found 40 off-target sites for two gRNAs for editing of *BnFAD2* gene homologues in *B. napus*. In another study, Yang et al. (2018) reported 57 off-target sites while editing *BnCLV* gene homologues. The number of off-target sites increases when using a smaller seed sequence in gRNA, for example, less than 7 bp, in the genome for the off-target effects.

Designing gRNAs with the least or no off-targets in polyploid crops becomes challenging. However, off-target effects can be avoided by selecting gRNAs with optimum GC content (50-60%) and relatively shorter gRNAs (18-20bp), avoiding mismatches and bulge formation in the 7-10 bp and 12 bp adjacent to PAM, respectively (Lin et al., 2014). Another strategy to reduce the off-target effects is to use a low concentration of gRNA/Cas9 complex. However, the concentration should not be too low that the on-target efficiency is also compromised (Jia and Wang, 2014). Hence, choosing a suitable promoter for driving the expression of gRNA and Cas9 genes is of crucial importance. Direct delivery of CRISPR components, such as RNP complexes comprising gRNA and Cas9 protein, into plant cells can substantially reduce the off-target effects as they are not stably transformed into the plant genome (Subburaj et al., 2016; Ahmad et al., 2020). The plant’s endogenous system quickly degrades these RNP complexes after their function, reducing the chance of targeting and editing other unintended locations. Off-target effects can also be reduced by using high-fidelity SpCas9 variants such as eSpCas9(1.0), eSpCas9(1.1), and SpCas9-HF1 (Slaymaker et al., 2016; Wang et al., 2017). These modified versions of the Cas9 protein show fewer off-targets as they require a precise 20-nucleotide guide sequence for identifying and editing targets.

### 3.5 Low editing efficiency

The efficiency of genome editing tools, particularly in polyploid crops, has been found to be very low (Zaman et al., 2019b). Several factors affect editing efficiency, including the number of delivered gRNAs, the activity and expression levels of the gRNA and Cas9 protein, the GC content of gRNA, the secondary structure of gRNA, and the target environment (Abe et al., 2019). While a single gRNA, can induce frameshift mutation in a single gene, multiple gRNAs may generate large deletions between targeted sites, thereby increasing the chance of creating multiallelic knockout mutations. However, the co-expression of multiple gRNAs often results in low editing efficiency (Jansing et al., 2019). The use of native promoters for gRNA and Cas9 expression is associated with higher editing efficiencies and co-editing events in polyploid crops (Johansen et al., 2019; Li et al., 2020). However, in species where native promoter sequences have not been discovered, the promoter of other relative species can be used, although editing efficiency may be compromised. For example, in *Brassica* genome editing experiments, the Arabidopsis U3 and U6 promoters are being used (Zheng et al., 2020). Editing efficiency may also be improved by codon optimization of Cas9-coding sequences and the use of enhancers (Kusano et al., 2018; Brauer et al., 2020; Grützner et al., 2021; Zhang et al., 2022b).

The target environment also plays an important role in efficient genome editing. Genome bias has been observed for gRNAs, which edit one genome more effectively than the other one in polyploids, as in the case of simultaneous editing of multiple *BnaSVP* gene copies. A comparatively larger number of edits were obtained for copies present in the subgenome A rapeseed as compared to that in the subgenome C at all target sites (Ahmar et al., 2022). Huang et al. (2020) also observed this phenomenon of marked preference for a gRNA to edit a specific gene more efficiently than others. They designed a gRNA with high sequence similarity to the four gene homologues of the *BnFAD2* gene located on two different subgenomes, A and C. However, most of the editing was observed in the *BnFAD2.A5* gene present in the subgenome A only.

Other factors affecting editing efficiency in *Brassica*, such as the incubation period of explants on regeneration medium and the choice of explants, have also been investigated. Lawrenson et al. (2015) reported high editing efficiency in *B. oleracea* calli kept on regeneration media for seven weeks as compared to those kept for four weeks with no off-target effects. Mikami et al. (2015) also reported higher editing efficiencies in prolonged tissue culture conditions in the case of rice. Editing efficiency may also vary with the target explant used for transformations. Jeong et al. (2019) designed eight gRNAs and tested the editing efficiency of CRISPR/Cas9 and gRNA complex by delivering them into the protoplasts of *B. rapa* leaves and selected four gRNAs with higher editing efficiencies to transform into hypocotyl explants. Surprisingly, only one out of the four gRNAs exhibited editing in regenerated plants, and the gRNA with the highest editing efficiency in protoplast culture gave no mutations. This could be also due to differences in the insertion position of T-DNA, which might affect their expression.

### 3.6 Molecular characterization of the edited genes

Molecular characterization of targeted mutations in polyploid crops is also challenging compared to diploids. Medium-throughput PCR-based assays, such as cleaved amplified polymorphic sequences (CAPS), T7 endonuclease 1 (T7E1), high-resolution melting analysis (HRMA), and capillary electrophoresis, which are typically used for molecular characterization of edited genes in diploid crops, may not provide a detailed characterization of the edited alleles/genes in polyploids. High sequence depth and longer read lengths are required to distinguish between the number of edited alleles/genes, leading to increased costs and labour involved in the genotyping of polyploids (May et al., 2023). Sanger and Illumina high-throughput sequencing are commonly used for the characterization of the type and extent of mutation(s) in polyploid crops. Computational tools like tracking of indels by decomposition (TIDE) (Brinkman et al., 2014) and Inference if CRISPR edits (ICE; Conant et al., 2022) are being employed for the quantitative assessment of the type and extent of mutations in case of Sanger sequencing for crops with low to moderate ploidy levels (Park et al., 2017; Liu et al., 2019). To determine the number of co-edited alleles/genes, the amplicons, amplified by allele-specific primers, are first cloned into a sequencing vector followed by monoclonal sequencing using the Sanger platform. This technique can obtain long read lengths, accurately distinguishing between alleles/genes, and assessing the editing efficiency of multiple gRNAs within a single allele/gene (Li et al., 2017; Eid et al., 2021).

## 4 Conclusion

Although genome editing has become a tool of choice for gene functional studies and precision breeding, its applications are still limited to diploid crops due to factors such as genome complexity, gene redundancy, the challenge of designing efficient gRNA and finding allele-specific mutations. It is expected that developments, such as finding new Cas protein variants, and advanced analytical approaches to identify edits, are likely to expand the utility of this toolbox for genome editing in polyploid crops. Once complete genome information is available, CRISPR/Cas technology can be effectively used to develop genome-wide mutant libraries of *Brassica* crops, as done in other crops such as rice. Besides crop improvement, the domestication process of wild *Brassica* species can be accelerated using CRISPR/Cas genome editing by targeting domestication-related genes, as evident in wild rice and tomato. CRISPR-based gene editing technology has emerged as a powerful tool for precision agriculture and will be effectively utilized for enhancing yield, biotic/abiotic stress tolerance, improving quality and plant architecture, haploid induction, and *de novo* domestication of *Brassica* crops. It is hoped that regulatory standards related to gene-edited crops will also be reformed to facilitate their fast commercialization and public acceptance.

Aggrevating the changing climate are the biotic and abiotic factors which result in significant yields losses in field crops. In addition to yield losses, biotic factors including fungus, bacteria, viruses, nematodes and insect attacks also deteriorate the crop produce quality (Giudice et al., 2021). The changing climatic patterns (which includes early and long high temperature regimes, unexpected heavy rainfalls, cloud bursts, severe winters) coupled with drought, salinity and heavy metal toxicity altogether pose a severe threat to global agricultural production and food security (Rahman et al., 2022). Therefore, development of stress-resilient crops is the need of the hour to ensure global food security and meet the UN’s target of a 60-70% increase in the agricultural crop production by 2050. CRISPR/Cas9 technology can be effectively employed to develop stress resilient crops by targeting genes involved in these stress related pathways. The resistance or tolerance to these stresses can be incorporated in two ways i.e., by knocking those genes in which are involved in resistance (R genes) or by knock down of genes involved in crop’s susceptibility (S genes). A more advanced CRISPRa (CRISPR activation) of R genes and CRISPRi (CRISPR interference) of S genes can also be employed to incorporate stress resilience in crop plants (Zafar et al., 2020). One of the main reasons for the crop failure due to sudden disease outbreak or changing environmental conditions is their minimized genetic diversity which have been lost in the process of intensive selection and domestication. Therefore, synthetic directed evolution (SDE) presents a promising solution to increase the localized sequence diversification of specific genes (e.g., stress related genes) and then selection of gene variants exhibiting better resistance to biotic and abiotic stresses (Zhang and Qi, 2019; Rao et al., 2021).

## Author contributions

NA, M-UR, and RG designed the study; NA and SF wrote the first draft; MM, QZ, RA, WZ, M-UR, and RG critically revised the study. All authors contributed to the article and approved the submitted version.
